# Genes of ACYL CARRIER PROTEIN Family Show Different Expression Profiles and Overexpression of ACYL CARRIER PROTEIN 5 Modulates Fatty Acid Composition and Enhances Salt Stress Tolerance in *Arabidopsis*

**DOI:** 10.3389/fpls.2017.00987

**Published:** 2017-06-08

**Authors:** Jiexue Huang, Caiwen Xue, Han Wang, Lisai Wang, Wolfgang Schmidt, Renfang Shen, Ping Lan

**Affiliations:** ^1^State Key Laboratory of Soil and Sustainable Agriculture, Institute of Soil Science, Chinese Academy of SciencesNanjing, China; ^2^University of Chinese Academy of SciencesBeijing, China; ^3^Institute of Plant and Microbial Biology, Academia SinicaTaipei, Taiwan

**Keywords:** acyl carrier proteins, salt stress, fatty acids, *Arabidopsis*, abiotic stress

## Abstract

Acyl carrier proteins (ACPs) are a group of small acidic proteins functioning as important cofactors in the *de novo* synthesis of fatty acids. In *Arabidopsis*, ACPs are encoded by a small gene family comprising five plastid members, *AtACP1* to *AtACP5*, and three mitochondrial members. The biological functions and the transcriptional responses to abiotic stresses of most AtACPs have yet to be elucidated. The present study extends previous findings and provides new knowledge on the function of ACPs by examining the responses of AtACP-encoding genes to several abiotic stresses and, in particular, the role of *AtACP5* in the adaptation to salt stress. Phylogenetic analysis showed that AtACP1, AtACP2, AtACP3, and AtACP5 can be classified into one group and separated from a group comprising AtACP4 and ACP homologs from related species. Quantitative RT-PCR analysis revealed that the expression of *AtACP1, AtACP2*, and *AtACP3* was induced by drought. Both iron deficiency and nitrogen starvation resulted in down-regulation of *AtACP4*. The most pronounced response was observed for *AtACP5*, the expression of which was dramatically decreased by salt stress. Knock-out of *AtACP5* showed increased sensitivity to NaCl stress, whereas transgenic lines overexpressing *AtACP5* displayed increased salt tolerance relative to the wild-type. Overexpression of *AtACP5* further led to an altered composition of fatty acids, mainly a decrease of oleic acid (C18:1) and an increase of palmitic acid (C16:0), and to a lower Na^+^/K^+^ ratio when compared to the salt stressed wild-type. The comprehensive transcriptional information on the small plastid AtACP gene family in response to various abiotic stresses and the further investigation of the *AtACP5* indicate that *AtACP5* might be critical for salt tolerance through alterations of the composition of fatty acids and, subsequently, the Na^+^/K^+^ ratio.

## Introduction

Due to their sessile life style, plants are unavoidably exposed to environmental stresses throughout their life cycle. To deal with such constraints, plants have evolved mechanisms that allow for efficient adaption to an ever-changing environment. Fatty acids (FAs) are pivotal constituents of cellular membranes, suberin, and cutin waxes that provide structural barriers to the environment (Beisson et al., 2007). Cellular membranes are a major target of environmental signals, and the maintenance of their integrity, permeability, fluidity and functionality of transport proteins by recalibrating lipid composition is critical for the resistance to different stresses (Rodriguez-Vargas et al., 2007; Huynh et al., 2012; Maejima et al., 2014).

Acyl carrier proteins (ACPs) play a central role in *de novo* FA synthesis. Fatty acid synthases (FASs) can be separated into two distinct classes. TypeI FASs consist of a single, large, multifunctional polypeptide and are common to mammals, fungi, and some bacteria. Type II FASs are found in archaea, bacteria and plants and are characterized by the involvement of discrete, mono-functional enzymes for FA synthesis. ACP exists as a separated domain (Jenke-Kodama et al., 2005). Plant ACPs are small (9 kD) separated polypeptides with 70–80 mostly acidic residues, modified by the covalent attachment of 4′-phosphopantetheine to a centrally localized serine (Wakil et al., 1983; Marrakchi et al., 2002). *De novo* biosynthesis of FAs proceeds via a conserved set of reactions, which are carried out during the elongation cycle (Smith and Sherman, 2008). ACPs are central components of FASs, which covalently bind all fatty acyl intermediates. In the first step, ACP synthase (ACPS) attaches a phosphopantetheine group from CoA on a serine residue of ACP in a conserved Asp-Ser-Leu motif to form holo-ACP (Mofid et al., 2002). The initial substrate of FASs, malonyl-CoA, is transferred to ACP and the acetyl-CoA unit (C2) is expanded to a butyryl group (C4). The synthetic cycle is repeated multiple times until saturated C16 or C18 acyl-ACPs are produced for utilization in membrane biosynthesis (Chan and Vogel, 2010).

Besides the production of saturated FAs (SFAs), ACPs are also involved in the biosynthesis of unsaturated FAs (UFAs), which proceeds via a slightly different reaction scheme. In *Escherichia coli*, most of the C_10_ acyl chain substrates are processed by β-hydroxyacyl dehydratase (FabZ) to form *trans*-2-decenoyl-ACP. Subsequently, enoyl reductase (FabI), β-Oxoacyl synthase I (FabB) or β-Oxoacyl synthase II (FabF) catalyzes the formation of SFAs. Alternatively, this fatty acyl intermediate can be converted through dehydration and isomerization to *cis*-3-decenoyl-ACP catalyzed by β-hydroxyacyl dehydratase (FabA) (Heath and Rock, 1996; Leesong et al., 1996), which can be further extended to UFAs by FabB (Feng and Cronan, 2009). Therefore, in *E. coli* FabBs and FabIs compete for *cis*-3- and *trans*-2-decenoyl-ACP, respectively, and the relative abundance of these two enzymes determines the ratio of saturated to UFAs in the membrane. The vast majority of UFAs in *E. coli* are *cis*-9-hexadecenoic acid (palmitoleic acid) and *cis*-11-octadecenoic acid (vaccenic acid) (Cronan, 1967; Morein et al., 1996).

The levels of UFAs can be changed to adjust membrane lipid fluidity to environmental conditions, mainly by regulating the activity of FA desaturases. For example, enhancement of polyunsaturated FA levels in chloroplast lipids was shown to be an effective means to resist chilling stress, while an opposite trend was observed during heat stress (Iba, 2002). Free linolenic acid itself is a stress signal and the precursor for phyto-oxylipin biosynthesis (Blee, 2002). Increasing evidence suggests that chloroplast oleic acid (18:1) levels are critical for normal pathogen defense responses in *Arabidopsis*, including programmed cell death and systemic acquired resistance (SAR) (Kachroo et al., 2001). Also, the *Arabidopsis* FAD2, an endoplasmic reticulum (ER)-localized ω-6 desaturase, which converts oleic acid (18:1) to linoleic acid (18:2) by inserting a double bond at the ω-6 position, is required for chilling and salt tolerance (Miquel et al., 1993; Zhang et al., 2012). All higher plants studied so far contain multiple isoforms of ACP, some of which are expressed ubiquitously while others appear to be expressed in a tissue-specific manner (Battey and Ohlrogge, 1990; Hlousekradojcic et al., 1992). The genome of the model plant *Arabidopsis thaliana* harbors three genes encoding mitochondrial ACPs, namely *mtACP1* (At2g44620), *mtACP2* (At1g65290), and *mtACP3* (At5g47630), and five plastidial ACPs, *AtACP1* (At3g05020), *AtACP2* (At1g54580), *AtACP3* (At1g54630), *AtACP4* (At4g25050), and *AtACP5* (At5g27200) (Meyer et al., 2007). Based on protein analysis, *AtACP1*, *AtACP2*, and *AtACP3* are expressed in all tissues examined (Hlousekradojcic et al., 1992), whereas *AtACP4* is expressed predominantly in leaves and the mRNA levels are increased by light (Shintani, 1996; Bonaventure and Ohlrogge, 2002). *AtACP1* and *AtACP3* are regulated by the transcription factor WRINKLED1 (WRI1) and are required for the accumulation of triacylglycerols in *Arabidopsis* seeds (Ruuska et al., 2002; Maeo et al., 2009). The expression of both genes followed a bell-shaped pattern that increased through the early seed development stages and peaked between 8 and 11 days after flowering (Ruuska et al., 2002). *AtACP2* was associated with the cell cycle (Menges et al., 2002), and its expression was up-regulated in developing siliques (Moire et al., 2004) and in cytokinin receptor mutants (Rashotte et al., 2003). *AtACP4 was* required to perceive the mobile SAR signal in distal tissues of *Arabidopsis* (Xia et al., 2009). Except for AtACP5, all plastid AtACPs showed increased protein abundance upon phosphate deficiency (Lan et al., 2012).

Generally, the expression levels of *ACPs* relate to the FA composition. Overexpression (OE) of *AtACP1* resulted in decreased 16:3 and increased 18:3 FAs (Branen et al., 2001). Compromised expression of *AtACP4* by both antisense *AtACP4* and T-DNA insertion led to reduced proportion of 16:3 FAs (Branen et al., 2003; Ajjawi et al., 2010) and caused a chlorotic phenotype and compromised photosynthetic competence (Ajjawi et al., 2010). Although all *Arabidopsis* ACP isoforms have been characterized except AtACP5, little is known regarding their putative involvement in the acclimation to abiotic stresses. Understanding the role of plastidial ACPs in environment stresses in general, and the molecular function of AtACP5, are the two main aims of the present study. We here described the transcriptional responses of five plastidial *ACPs* to various abiotic stresses such as salt, drought and deficiencies in nitrogen, phosphorus, potassium, and iron in *Arabidopsis*. It was found that the expression of *AtACP1-3* was induced by drought, *AtACP4* was down-regulated in nitrogen- and iron-deficient plants, and *AtACP5* was dramatically inhibited by salt stress. Loss of *AtACP5* function resulted in hypersensitivity to salt stress, while *AtACP5* OE lines showed increased salt tolerance. The results showed that alterations in FA composition were causative for the different phenotypes observed under salt stress. Specifically, decreased oleic acid and increased palmitic acid levels in *AtACP5* OE plants were shown to contribute to increased salt tolerance, possibly by maintaining the cellular homeostasis of Na^+^ and K^+^.

## Materials and Methods

### Plant Material and Treatments

Plants were grown in a growth chamber on agar medium as described by Estelle and Somerville (1987). Seeds of *A. thaliana* Col-0 accession and the T-DNA insertion mutant *atacp5* (SALK_111501C) were ordered from NASC^1^. Seeds were surface sterilized with 70% ethanol and bleach (0.5% NaClO + 0.5% Tween) and sown on solid agar plates containing ES medium (Estelle and Somerville, 1987). The medium was composed of (mM): KNO_3_ (5), MgSO_4_ (2), Ca (NO_3_)_2_ (2), and KH_2_PO_4_ (2.5), (μM): H_3_BO_3_ (70), MnCl_2_ (14), ZnSO_4_ (1), CuSO_4_ (0.5), NaCl (10), Na_2_MoO_4_ (0.2), and Fe(III)-EDTA (40), solidified with 0.8% agar, 1% sucrose, and 4.7 mM MES. The pH was adjusted to 5.5. Plates were vernalized in darkness for 2 days at 4°C and then transferred to a growth chamber at 22°C and 70% humidity under a 16-h-light/8-h-dark photoperiod.

To observe the seedling phenotype under salt stress, 50 seeds of each genotype were germinated and grown for 20 days on ES media supplemented with 0, 125 or 150 mM NaCl. The growth status of the plants was divided into four categories: non-germination, normal, sub-healthy, and dead. The percentage of each category was determined. For each treatment, all 50 seedlings were collected and weighed together, and the average fresh weight (FW) per seedling was calculated. Similar experiments were carried out with 5-day-old seedlings transferred to ES media supplemented with 0 or 125 mM NaCl for another 9 days.

To analyze the salt sensitivity of mature plant, 10-day-old seedlings were transferred to soil to grow for additional 11 days and then treated with NaCl solution either by stepwise increase or added once to the maximum of 250 mM NaCl. The average FW of rosette leaves per plant was calculated.

### Characterization of the T-DNA Insertion Mutant

Homozygosity of the mutants was determined by PCR from genomic DNA using gene-specific (LP: 5′-CTCAGAGATGAAGGATGCTGG-3′; RP: 5′-CCATCTCTCTCGATCAGATCG-3′) and T-DNA left border primers LBa1, and further analyzed by DNA sequencing to confirm the insertion site of the T-DNA in the gene.

### DNA Constructs and Plant Transformation

To generate the *AtACP5* OE construct (*35S::AtACP5*), the full-length ORF (420 bp) of the *AtACP5* gene was amplified with *AtACP5* specific primers (forward: 5′-CTAGGTACCATGGCGACAAGTTTCTGCT-3′, *KpnI*; reverse: 5′-CATCTGCAGCTAAGCAGTCTTCTCTTGGACG-3′, *PstI*), ligated into the same sites of the modified binary vector pCAMBIA2301 behind the cauliflower mosaic virus 35S promoter. The T-DNA construct was introduced into wild-type plants via Agrobacterium-mediated transformation as described previously (Clough and Bent, 1998).

### Total RNA Isolation and Quantitative Real-Time RT-PCR Analyses

Plant samples were collected in liquid nitrogen and stored at -80°C for RNA extraction. Total RNA was isolated according to the manufactures’ instructions using Trizol reagent (Invitrogen). One microgram total RNA was used for reverse transcription (TaKaRa, with gDNA Eraser, Cat#RP047A). The cDNA was diluted 12 times, and 2 μl were used as a template in a 10 μl PCR reaction. Real-time RT-PCR analysis was performed using SYBR Green Perfect mix (TaKaRa, Cat# RR420A) on a Thermo PIKOREAL 96 Real-Time PCR System, with a program of 40 cycles and the following conditions: 95°C for 5 s, 60°C for 30 s with *TUA3* (*tubulin alpha-3*, At5g19770) as endogenous control. The primers used in this study are listed in Supplementary Table 1.

### FA Composition

The FA composition in seedling tissues was determined as previously described (Li et al., 2006; Wang et al., 2012) with slight modification. Ten-day-old seedlings (0.15 g) of each genotype were heated in 2 ml 10% methanol-KOH at 80°C for 2 h. After the mixture was cooled to room temperature, 1 ml of 6 N HCl was added and the mixture was extracted twice with 2 ml of hexane. The extracts were dried under nitrogen stream. To this end, 1 ml of 5% (v/v) sulfuric acid in MeOH (freshly prepared for each use), 25 μl BHT solution (0.2% butylated hydroxy toluene in MeOH), 50 μg triheptadecanoin (as a triacylglycerol internal standard to generate methyl heptadecanoate), and 300 μl toluene as co-solvent were added to the tube. The mixture was vortexed for 30 s and then heated to 90–95°C for 1.5 h. After cooling to room temperature, 1.5 ml 0.9% NaCl (w/v) were added and fatty acid methyl esters (FAMEs) were extracted with 3 ml × 2 ml hexane. Pooled extracts were evaporated under nitrogen and then dissolved in 400 μl hexane. The FAME extracts were analyzed by gas chromatography (GC). GC analysis was performed on a Thermo GC Ultra with a flame ionization detector (FID) on a DB23 column (30 m by 0.25 mm i.d., 0.25 μm film; J&W Scientific, Folsom, CA, United States). The commercial standard FAME mixture (Sigma, CRM47885) was estimated quantitatively. The GC conditions were: split mode injection (1:40), injector and FID temperature, 260°C; oven temperature program, 150°C for 3 min, then increasing at 10°C/min to 240°C and holding this temperature for 5 min.

### Determination of the Na^+^ and K^+^ Concentrations

Ten-day-old seedlings grown on ES media supplemented with or without 125 mM NaCl were used for cation concentration determination as previously described (AOAC, 1990). Seedlings were harvested, rinsed with deionized water, and dried at 70°C for 2 days. One-hundred milligrams of ground dry matter was then extracted with 20 ml deionized water by boiling for 2 h. The extract was filtered and the volume was set to 25 ml. Na^+^ and K^+^ concentrations were determined by flame spectrophotometry using a standard curve (coefficients > 0.99) made by standard Na^+^ and K^+^ solutions, and the relative Na^+^ and K^+^ concentrations were calculated as mg/g DW.

## Results

### *Arabidopsis* Plastidial ACPs Are Phylogenetically Divided into Two Clades

**FIGURE 1:**
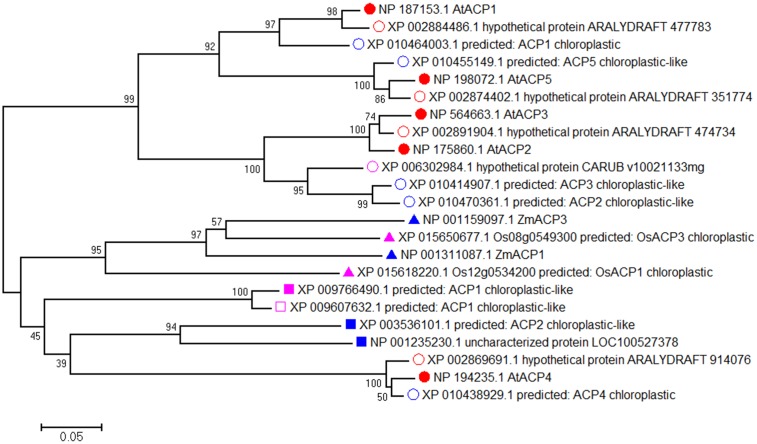
The phylogenetic tree of AtACP1-5 proteins. A phylogenetic tree was conducted using full-length maize (*Zea mays*, blue triangle), rice (*Oryza sativa*, purple triangle), soybean (*Glycine max*, blue quadrate) and tobacco (*Nicotiana sylvestris*, purple quadrate; *Nicotiana tomentosiformis*, purple unfilled quadrate) and other species (*Arabidopsis lyrata* subsp. *lyrata*, red unfilled circular; *Camelina sativa*, blue unfilled circular; *Capsella rubella*, purple unfilled circular) with the highest similarity amino acid sequences related to AtACP1-5 from *Arabidopsis* (red filled circular) by MEGA6.06 software using neighbor-joining method. The numbers beside each node represent bootstrap values based on 1,000 replications. The scale bar indicates the relative amount of change along branches.

To investigate the divergence between *Arabidopsis* ACPs and ACPs in other species, we performed a protein BLAST search with the amino acid sequences of AtACP1-AtACP5. Highest scores (similarities of more than 90%) were obtained for Brassicaceae species such as *Arabidopsis lyrata* subsp. *lyrata* and *Camelina sativa* (Supplementary Table 2). Extending the search for related proteins in the genomes of the monocotyledonous species rice (*Oryza sativa*) and maize (*Zea mays*) and the dicotyledons soybean (*Glycine max*) and tobacco (*Nicotiana tabacum*) yielded relatively low similarities of the amino acid sequences (less than 50%). We then analyzed the phylogenetic relationships of the orthologous ACPs using the MEGA6.06 software package (**Figure 1**). This analysis subdivided the proteins into two clades with AtACP1, AtACP2, AtACP3, AtACP5 and most ACPs from Brassicaceae species comprising one clade, and AtACP4 and ACPs from species unrelated to Brassicaceae forming a second clade, suggesting that the divergence between AtACP4 and the other four AtACPs appeared early in evolution. In the first clade, AtACP2, AtACP3, and some AtACP2/3-like protein from other Brassicaceae species form a subgroup. The high homology between AtACP2 and AtACP3 (similarity 92.6%) and clusters of arrangement in the Chr 1 suggested that these genes were created by a local gene duplication. AtACP1 and AtACP5 showed moderate similarity (67.9%) and fell into another group. Multiple alignment of these ACPs by DNAMAN revealed that the highly conserved amino acid sequence LGADSLDTVEIVM include the serine residue to which the cofactor 4′-phosphopantetheine is attached (Supplementary Figure 1). These results suggest that the five plastidial *Arabidopsis* ACPs contain a highly conserved motif that may play similar molecular roles in different processes.

**FIGURE 2:**
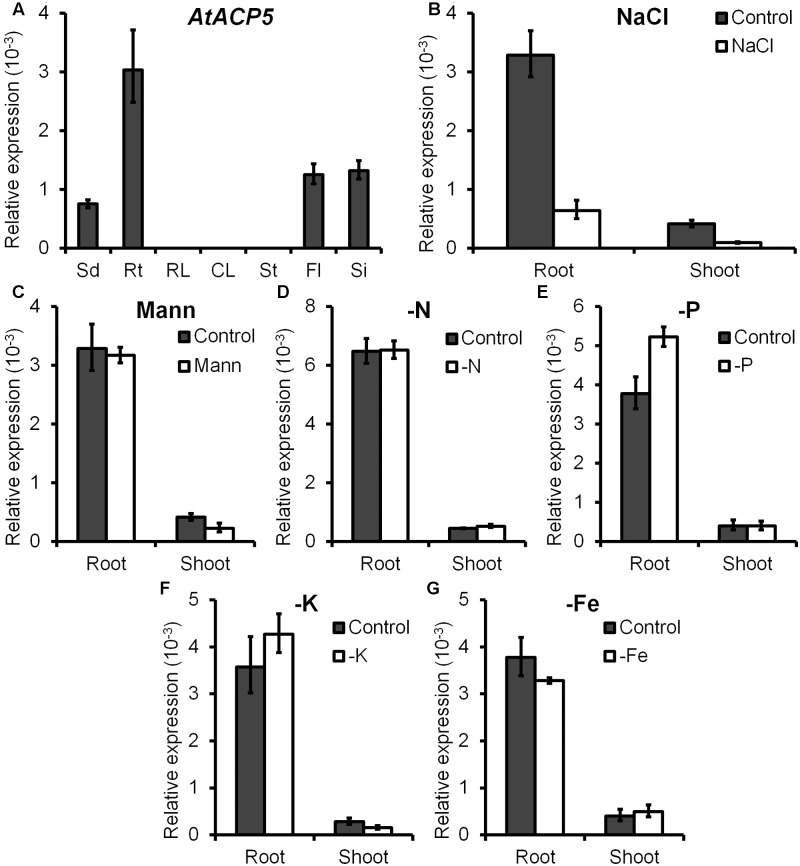
*AtACP5* is preferentially expressed in root and chiefly repressed under NaCl treatment. Expression analyses of *AtACP5* in the wild-type Col-0 plants. **(A)** Quantitative real-time RT-PCR (qRT-PCR) analyses of *AtACP5* transcripts. Total RNA was extracted from 7-day-old seedlings (Sd), roots of 14-day-old seedlings grown on ES agar media (Rt), and rosette leaves (RL), cauline leaves (CL), stems (St), flowers (Fl), and young siliques (Si) of 5-week-old plants grown on nutrition soil. **(B–G)** qRT-PCR analysis of *AtACP5* upon treatment with different stresses, including 150 mM NaCl, 300 mM mannitol, nitrogen-, phosphorus-, potassium-, and iron-deficiency. Ten-day-old seedling were grown on ES media and total RNA was extracted from shoots and roots after 3-day-treatment. Expression values were calculated using 2^-ΔCT^ method with *TUA3* (*tubulin alpha-3*, At5g19770) as endogenous control. Data represents the average of three independent experiments ±SD.

### *AtACP5* Is Preferentially Expressed in Roots and Repressed under NaCl Treatment

To decipher the biological function of the five plastidial ACPs in *Arabidopsis*, we first examined the expression patterns of these genes in wild-type plants grown under normal and various abiotic stress conditions by quantitative real-time RT-PCR (qRT-PCR). Results showed that expression of *AtACP5* was detected in all test tissues including seedlings, roots, flowers, and siliques but not in rosette leaves, cauline leaves and stems, with being highest in roots (**Figure 2A**). Consistent with previous protein analyses, transcripts of *AtACP1*, *AtACP2*, and *AtACP3* were present in all tissues (Supplementary Figure 2) (Hlousekradojcic et al., 1992). Among them, *AtACP4* was observed to be the most abundant gene, which was also expressed across all tissues. In line with previous reports (Shintani, 1996), *AtACP4* mRNA was most abundant in cauline leaves, rosettes and seedlings (Supplementary Figure 2). To investigate the expression responses of *AtACPs* to various abiotic stresses, plants were subjected to various treatments followed by qRT-PCR analysis. Upon treatment with 150 mM NaCl, the transcript level of *AtACP5* was reduced to 20% of the value observed under control conditions (**Figure 2B**). However, no significant differences in expression upon other various abiotic treatments were observed for *AtACP5* (**Figures 2C–G**). These results suggested *AtACP5* might be involved in salt stress process. Expression levels of *AtACP1*, *AtACP2*, and *AtACP3* in shoots were up-regulated by drought (300 mM mannitol) (Supplementary Figure 3B), while transcript level of *AtACP4* was reduced by nitrogen and iron deficiency (Supplementary Figure 3). These results confirmed that the five AtACPs respond differently to various abiotic stresses and could be associated with multiple processes.

### Overexpression of *AtACP5* Enhances Salt Stress Tolerance

**FIGURE 3:**
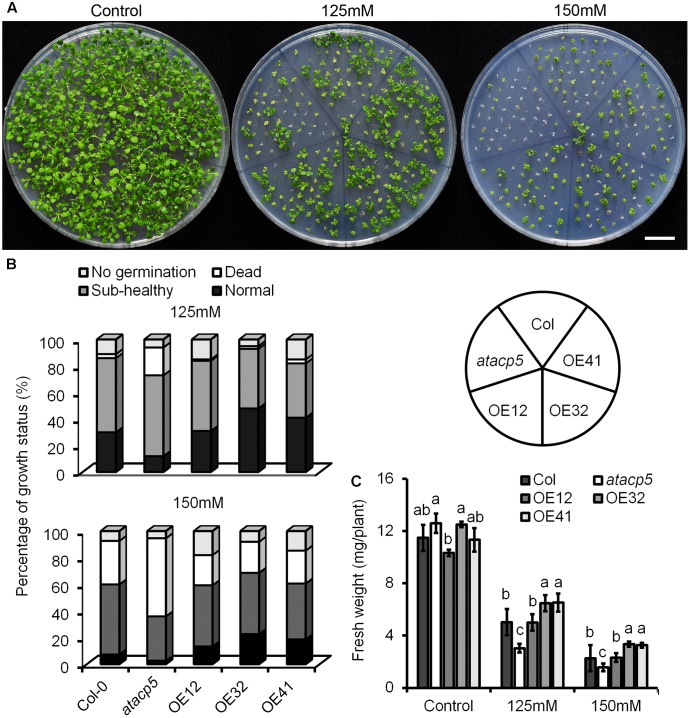
NaCl hypersensitive phenotypes of the *atacp5* mutant. **(A)** Plates with 20-day-old seedlings of Col-0, *atacp5*, OE12, OE32, and OE41 grown in Petri dishes with ES media supplemented with 0 mM (control), 125 mM or 150 mM NaCl. Growth status and survival rate of the salt-stressed seedlings. **(B)** For each treatment, three plates each containing 50 seedlings for each genotype were scored. Black column represents percentage of normal seedlings, dark gray column represents percentage of sub-healthy ones with yellowish cotyledons, white column represents percentage of white and dead seedlings, and gray column represents ungerminated seeds. **(C)** Statistical analysis of the fresh weight of whole plant. Illustrated are the mean values ±SD (*n* = 3). Data from one of two experiments with similar results are shown. Different letters represent significantly different values at *P* < 0.05 (Duncan’s multiple range test). Scale bar = 2 cm **(A)**.

Since *AtACP5* specifically responded to salt stress, we therefore further explored a potential function of AtACP5 in the salt stress response using a genetic approach. The mutant SALK_111501C carrying a T-DNA insertion in the second intron of *AtACP5* was identified and confirmed to be dramatically reduced at mRNA level, herein named as *atacp5* (Supplementary Figure 4). Overexpression lines harboring a *35S::AtACP5* construct were generated, and three homozygous OE lines designated as AtACP5-OE12, AtACP5-OE32, and AtACP5-OE41 with massively increased expression levels were selected for further experiments (Supplementary Figure 4). Twenty-day-old seedlings of the wild-type, *atacp5* mutant plants and the three OE lines were grown on ES media supplemented with 0, 125 or 150 mM NaCl for growth analysis (**Figure 3A**). Growth was divided into four categories: non-germinating, normal (green leaves), sub-healthy (yellowish-green leaves), and dead (chlorophyll bleaching). Under control conditions (ES media), no differences in germination rate and growth status were observed between the three genotypes. However, growing plants on media containing 125 mM NaCl significantly increased the percentage of dead *atacp5* mutant plants (21%) when compared with wild-type plants (3%) and OE lines (1–3%). The percentage of normal seedlings listed in descending order were 31–48% for OE lines, 30% for wild-type plants, and 12% for *atacp5* mutants. Raising the NaCl concentration to 150 mM decreased growth of all genotypes under investigation. For seedlings carrying the *35S::AtACP5* construct, 13–22% were classified as normal, a rate that was significantly higher than that of the wild-type (**Figure 3B**). By contrast, among *atacp5* mutant plants less than half normal or sub-healthy seedlings were counted relative to wild-type plants. A similar picture was observed with respect to the FW. While under control conditions no significant differences were observed between the genotypes, OE lines grew larger and gained more FW than wild-type plants on both high-salt media; *atacp5* plants produced the lowest FW during the experimental period (**Figure 3C**). Five-day-old seedlings transferred to ES media supplemented with 0 or 125 mM NaCl and grown for 9 days showed similar results (Supplementary Figure 5).

**FIGURE 4:**
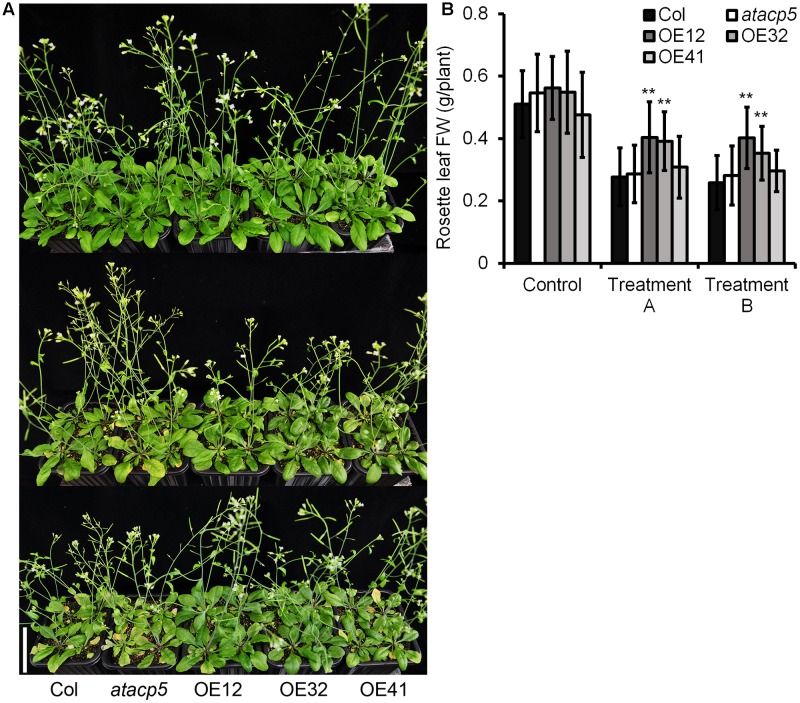
NaCl tolerance phenotypes of the overexpressing *AtACP5*. Wild-type Col-0, mutant *atacp5*, three overexpression transgenic lines OE12, OE32, and OE41 were used in assessing salt tolerance. Each plant line each set of 60 plants was divided into three groups (Control, treatment A, and treatment B) of 20 plants each. We applied 25 ml water every other day over the 16-day watering treatment. The control group received no NaCl supplementation. The remaining groups were watered with NaCl solution. Treatment A: the concentrations of NaCl supplementation were increased stepwise by 83.3 mM every other days for each line, to the indicated maximum 250 mM NaCl; Treatment B: salt solution was added once to the indicated maximum 250 mM NaCl. **(A)** Photographs after 16-day salt treatment (upper, control; middle, treatment A; down, treatment B). **(B)** Statistical analysis of the fresh weight of the rosette leaves. Illustrated are the mean values ± SD (*n* = 20). Data from one of two experiments with similar results are shown. Statistical analysis was performed by using an unpaired two-tailed Student’s *t*-test: ^∗∗^*P* < 0.01. Scale bar = 5 cm **(A)**.

To assess the sensitivity of mature plants to salt stress, we transferred 10-day-old seedlings from media to soil for additional 11 days with subsequent NaCl treatment (**Figure 4A**). While wild-type and mutant plants displayed progressive chlorosis, reduced leaf size and general growth inhibition when watered with a NaCl-containing solution, significantly higher FW of rosette leaves from OE lines relative to the wild-type indicated increased tolerance to NaCl (**Figure 4B**).

### Overexpression of *AtACP5* Results in Decreased Oleic and Increased Palmitic Acid Levels under Salt Stress

**FIGURE 5:**
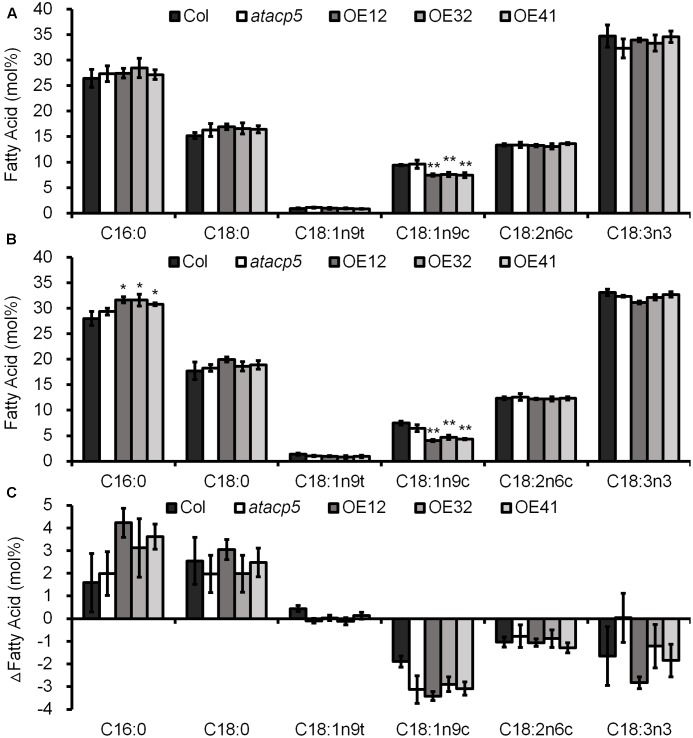
Overexpressing *AtACP5* caused decreased seedlings C18:1n9c fatty acids. Fatty acid analyses of wild-type Col-0, mutant *atacp5*, and three overexpression transgenic lines OE12, OE32, and OE41. Ten-day-old seedlings germinated and grown in Petri dishes with ES media supplemented with 0 (control, **A**), 125 mM NaCl **(B)**. The ΔFatty acid (difference value NaCl versus control treatment, **C**). Error bars represent SD values for three biological replicates (each replicate consisted of 50 seedlings). Statistical analysis was performed by using an unpaired two-tailed Student’s *t*-test: ^∗^*P* < 0.05, ^∗∗^*P* < 0.01.

Acyl carrier protein is a critical cofactor for FA biosynthesis. In order to investigate the effect of AtACP5 on FA levels, we analyzed the major FA species in 10-day-old seedlings by GC. Analysis of FA composition revealed that in OE lines the levels of monounsaturated FAs (oleic acid, C18:1n9c) under both control condition and salt stress decreased by approximately 20 and 40%, respectively, whereas the levels of SFAs (palmitic acid, C16:0) increased under salt stress compared to wild-type plants (**Figures 5A,B**). No differences in patterns were observed for seedlings FAMEs in the *atacp5* mutant lines compared to Col-0, probably due to the fact that this gene product is primarily limited in root and low abundance. Notably, the percentage of SFAs (C16:0 and C18:0) increased differentially between the two growth types, whereas the percentage of UFAs (C18:1, C18:2, and C18:3) decreased in all plants under salt stress relative to control conditions (**Figure 5C**). This suggests that increased levels of SFAs (C16:0) and reduced concentrations of UFAs (C18:1) improved the salt tolerance of OE lines.

### OE Lines Accumulate Less Na^+^ under High-Salt Conditions

**FIGURE 6:**
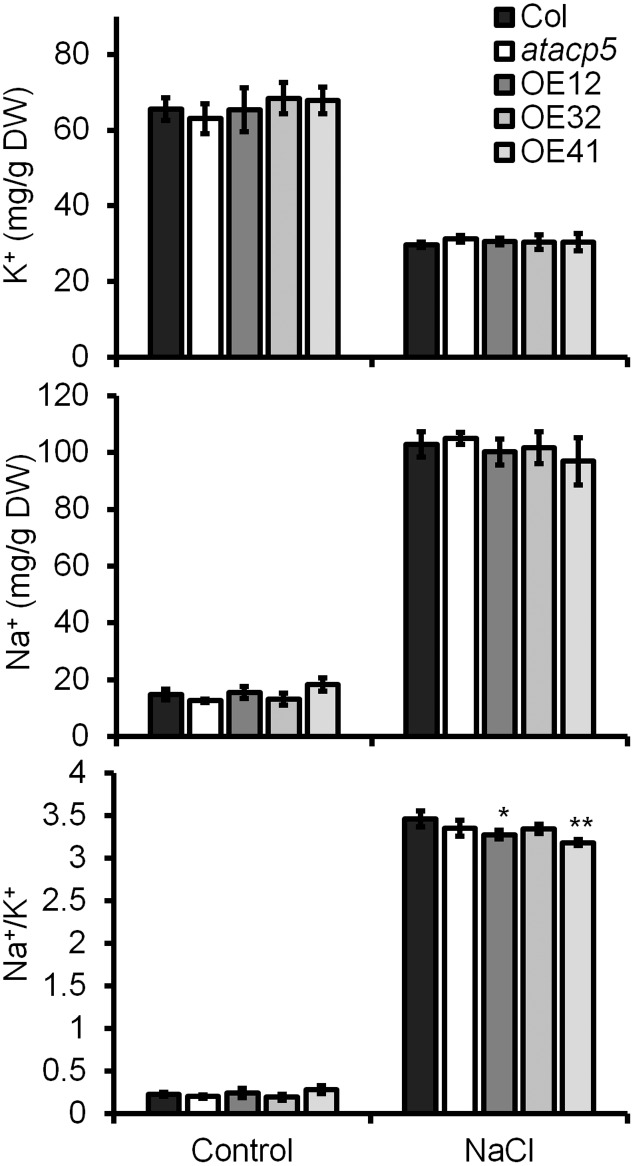
Overexpression (OE) lines accumulates less Na^+^ relative to Col-0 and *atacp5* in media supplemented with NaCl. Ten-day-old seedlings grown on ES media supplemented without (control) or with 125 mM NaCl (NaCl) were used. Results are presented as means and standard errors from three independent experiments. Statistical analysis was performed by using an unpaired two-tailed Student’s *t*-test: ^∗^*P* < 0.05, ^∗∗^*P* < 0.01.

The cytosolic Na^+^/K^+^ ratio is a key determinant of plant salinity tolerance. To investigate whether the maintenance of cellular ion homeostasis contributes to improved tolerance of *AtACP5* OE lines, we compared the Na^+^ and K^+^ concentrations of the wild-type, *atacp5* mutant plants and three OE lines. No significant differences in the Na^+^ and K^+^ concentration were detected between the genotypes when seedlings were grown in the absence of NaCl (**Figure 6**). Upon treatment with 125 mM NaCl, OE lines accumulated less Na^+^ than the wild-type while K^+^ levels were maintained, leading to a significantly decreased Na^+^/K^+^ ratio in OE lines (**Figure 6**).

## Discussion

Acyl carrier proteins are a family of universal, relatively abundant and highly conserved carriers of acyl, representing the core of the FAS system. Most species encode at least one ACP, but multiple isoforms and even dozens of homologous carrier proteins involved in FAS and secondary metabolism have been reported, suggesting functional diversity. For instance, more than 85 ACPs are encoded in the genome of *Streptomyces avermitilis* (Ikeda et al., 2003), the soybean (*G. max*, complex allotetraploid) genome harbors 25 ACPs (Wang et al., 2014). *Arabidopsis*, with its relatively simple genome, encodes five plastidial and three mitochondrial ACPs (Meyer et al., 2007). Phylogenetic analysis of 517 putative ACP proteins from 43 plant species identified a legume-specific subclade (Wang et al., 2014), supporting the assumption of functional diversification.

In the present investigation, phylogenetic analysis of *Arabidopsis* ACPs, regardless using neighbor-joining, maximum likelihood or minimum-evolution methods, revealed a subclade comprising all *Arabidopsis* plastidial ACP proteins (except AtACP4) and putative ACPs from other *Brassicaceae* (**Figure 1**), indicating divergence between different families during evolution. The formation of another subclade of AtACP4 suggests that AtACP4 and ACPs from other species unrelated to Brassicaceae might be derived from a different ancient gene. Apart from gene duplication and *de novo* gene generation, horizontal gene transfer (HGT) is an important way of acquiring new genes. Most HGT events have been occurred in prokaryotes (Gogarten et al., 2002) and unicellular eukaryotes (Keeling and Palmer, 2008), while the HGT is also involved in the evolution of high plant (Bock, 2010). The first example of nuclear HGT in seed plant is a Mu-like element (MULE) transposon between in Setaria species (millets) and in the rice (*O. sativa*) (Diao et al., 2006). Taken together, the exact mechanism of AtACP4 evolution awaits more evidence. Nevertheless, all *Arabidopsis* ACPs display the basic features of ACP proteins. Sequence alignment with homologous ACP proteins identified the characteristic and highly conserved LGADSLDTVEIVM motif comprising the DSL motif (Supplementary Figure 1), which represents the recognition site for ACPs (Mofid et al., 2002).

In plants, ACPs are expressed both ubiquitously and in a tissue-specific manner (Battey and Ohlrogge, 1990; Hlousekradojcic et al., 1992). The presence of multiple ACP isoforms and their differential responsiveness to external stimuli indicate that individual ACPs may play different roles in the adaptation to environmental conditions. For example, it was shown that the expression of *AtACP4* was induced by light, whereas the abundance of *AtACP2* or *AtACP3* mRNA was not responsive to the light regime (Bonaventure and Ohlrogge, 2002). In the present study, we found that the expression of *AtACP4*, but not of the other ACPs under study was down-regulated by nitrogen and iron deficiency (**Figure 2D**). *AtACP4* antisense plants (Branen et al., 2003) and T-DNA insertion mutants (*acp4-1* and *acp4-2*) showed a different degree of leaf bleaching appearance, reduced photosynthetic efficiency, and a decreased concentrations of 16:3 FAs. The lower concentrations of 16:3 FAs are attributed to the lower concentration of 16:3 FAs on the monogalactosyldiacylglycerol (MGDG) moiety, and most of the 16:3 FAs in *Arabidopsis* leaf tissue are found on the sn-2 position of MGDG (Ajjawi et al., 2010). *AtACP4* thus is a major player controlling the biosynthesis of FAs for chloroplast membrane lipids. Similarly, plant experiencing nitrogen or iron deficiency developed similar chlorotic phenotypes. Iron deficiency has been well documented to impair chlorophyll biosynthesis and chloroplast development (Mori, 1999), while N-starvation changes the chloroplast structure associated with alterations in thylakoid membrane structure and degradation of plastidial membrane lipids, leading to a damaged photosynthetic systems (Siaut et al., 2011; Wang et al., 2011). These results suggest that the expression level of *AtACP4* positively correlated with the photosynthetic systems status, and probably functions as a hub among light, nitrogen and iron deficiency. The expression of *AtACP5* was significantly repressed under salt stress (**Figure 2B**); while no significant differences in transcript levels were observed under other stresses, especially under drought stress (**Figures 2C–G**). These results thus indicated that the down-regulated expression of *AtACP5* under salt stress might be due to ion toxicity rather than osmotic stress. Consistent with these results, a knock-out T-DNA insertion mutant exhibited increased salt sensitivity whereas *AtACP5* OE lines showed increased tolerance to salt treatment (**Figures 3, 4**), indicating a possible role of *AtACP5* in salt stress tolerance.

*AtACP5* OE lines accumulated more palmitic acid 16:0 and less oleic acid 18:1 FAs compared with wild-type plants (**Figures 5A**, B). The major fate of 16:0 and 18:l acyl chains produced in the plastid is to form the hydrophobic portion of glycerolipid molecules, which are components of all cellular membranes (Ohlrogge and Browse, 1995). Surprisingly, *atacp5* loss-of-function did not contain less FAs, which could be expected from the observed increase in the OE lines. One possible reason is genetic redundancy; the *Arabidopsis* genome harbors five plastidial ACPs carrying a highly conserved consensus motif. Overexpression of *AtACP1* resulted in decreased concentration of 16:3 and increased levels of 18:3 FAs (Branen et al., 2001); inhibiting the expression of *AtACP4* led to reduced 16:3 FA concentration (Branen et al., 2003; Ajjawi et al., 2010). These genes may function in concert with *AtACP5* to regulate FA homeostasis. An alternative explanation is that the modification of FA composition is local rather than systemic. The detected FAs in the present study were from 10-day-old intact seedlings rather than from roots, which will dilute the contributions of the roots to the total FAs. Furthermore, *AtACP5* is primary expressed in root and could be functional mainly in the roots. In summary, although no significant differences in FA profiles were found between *atacp5* and the wild-type, the results from the *AtACP5* OE lines suggest that a complex regulatory network of FA metabolism exists in plants and *AtACP5* is a key player in FA homeostasis. Future study focusing on the root FA profiles among the three genotypes would expand our understandings toward the functions of *AtACP5* in plants.

Fatty acids are major components of membranes and as such part of the mechanisms by which cellular processes are adapted to environmental constraints. Changes in plasma membrane lipids are an adaptation mechanism to salinity in broccoli roots (Lopez-Perez et al., 2009). Studies in cucumber, soybean, and borage (*Borago officinalis L.*) have reported that salinity caused a significant increase of SFAs and UFAs in thylakoid membranes, plasma membrane, and leaf, respectively (Surjus and Durand, 1996; Jaffel-Hamza et al., 2013; Shu et al., 2015). Similarly, increased salinity was shown to reduce the plasma membrane fluidity in the halophyte *Spartina patens calli*, which was associated with increased FA saturation (Wu et al., 2005). The amount of 18:3 FAs was found to be reduced under salt stress in salt-tolerant, but not salt-sensitive citrus cells (Gueta-Dahan et al., 1997), wheat (*Triticum aestivum*) (Mohamed et al., 1994), and soybean (*G. max*) (Surjus and Durand, 1996). Due to their role in membrane rigidity, SFAs in the membrane bilayer reduces its permeability and fluidity (Van Blitterswijk et al., 1981). Moller et al. (2007) reported that reduction of polyUFAs in membranes contribute to salt tolerance via amelioration of the oxidative effects of salt. On the contrary, some studies reported that increasing UFAs resulted in salt tolerance. In *Synechococcus*, increasing the expression of the desA gene, which encodes the Δ12 acyl-lipid desaturase involving in the synthesis of diunsaturated FAs, enhanced NaCl tolerance due to an increased ability to repair the photosynthetic and Na^+^/H^+^ antiport systems (Allakhverdiev et al., 2001). Application of exogenous linoleic acid exhibited protective effects on tonoplast function in barley seedlings under salt stress (Zhao and Qin, 2005). An increase of UFAs in membrane lipids contributed to an alleviated salt-induced photoinhibition of PSII in *Thellungiella halophile* (Sui and Han, 2014). However, Chalbi et al. (2013), also revealed that the ability to increase unsaturation in FAs was not a determinant factor for salt resistance in barley species. Nevertheless, in *Arabidopsis*, the FA desaturases FAD2 and FAD6, which enhance the degree of unsaturation of FAs, were found to be required for salt tolerance (Zhang et al., 2009, 2012). ER-localized FAD2 and plastid-localized FAD6 encode two ω-6 desaturases that convert oleic acid (18:1) to linoleic acid (18:2) by inserting a double bond at the ω-6 position, FAD3 (ER) and FAD7 and FAD8 (plastids) encode three ω-3 desaturases, which convert linoleic acid (18:2) to linolenic acid (18:3) by inserting a double bond at the ω-3 position. Both *fad2* and *fad6* mutants showed increased cytoplasmic Na^+^ accumulation and enhanced sensitivity to salt stress due to increased 18:1 and decreased 18:2 FA levels. In agreement with the decreased drought tolerance of transgenic FAD7 antisense tobacco plants (Im et al., 2002), OE of either *FAD3* or *FAD8* increased tolerance to drought in transgenic tobacco plants (Zhang et al., 2005). Since increased 18:1 FAs resulted in increased sensitivity to salt, it is reasonable to assume that decreased amounts of 18:1 FA levels contribute to salt tolerance. In agreement with previous studies in *Arabidopsis* mentioned above, in the present study, although the ratio between SFAs and UFAs was increased under salt stress in all genotypes (**Figure 5C**), OE of *AtACP5* further led to an altered composition of FAs, mainly a decrease of oleic acid (C18:1) and an increase of palmitic acid (C16:0), which could explain why OE of *AtACP5* can increase salt tolerance. ACPs have been documented to be involved in the biosynthesis of both SFAs and UFAs (Heath and Rock, 1996; Leesong et al., 1996; Feng and Cronan, 2009; Chan and Vogel, 2010). Thus, manipulating the expression of ACPs could be a strategy to increase the tolerance of plants to environmental stresses including salt.

Under saline conditions, rapid influx of Na^+^ from the soluble phase of the soil into the cytoplasm of cortical cells in plant roots occurs through non-selective cation channels (NSCCs) rather than through the high-affinity K^+^ transporter HKT1 (Essah et al., 2003). The relatively high permeability for Na^+^ of many depolarization-activated NSCCs (DA-NSCCs) suggests they are also important contributors to the uptake and translocation of Na^+^, and thus their function impacts on salt tolerance. Increased 3′,5′-cyclic guanosine monophosphate (cGMP) affected monovalent cation homeostasis during salt stress and reduced Na^+^ uptake through NSCCs such as CNGC3 (Gobert et al., 2006). Some NSCCs respond to physical stimuli, relaying external signals into electrical and/or Ca^2+^ signals through the action of mechanosensitive channels (MCs) whose gating depends on changes in tension forces on the membrane. Maintenance of appropriate tension forces depends on membrane integrity and fluidity, which are largely affected by the composition of lipids in general and FAs in particular (Mikami and Murata, 2003). To maintain ion homeostasis, plants could secrete Na^+^ out of the cell via plasma membrane-bound Na^+^/H^+^ antiporters (e.g., SOS1) and sequester Na^+^ into the vacuole via Na^+^/H^+^ antiporters (e.g., NHX1) on the tonoplast (Wu et al., 1996; Apse et al., 1999). In the present study, no significant changes in the transcripts of *AtSOS1* and *AtNHX1* were detected (Supplementary Figure 6), suggesting that both secreting and sequestering Na^+^ might not be predominated among genotypes. However, Na^+^ concentration was slightly reduced while K^+^ concentration was unchanged in OE lines, leading to a lower Na^+^/K^+^ ratio, which might confer OE lines more tolerant to salt stress. The exact mechanisms by which overexpressing *AtACP5* increases salt tolerance awaits further study, although it could be possible that ectopic expression of *AtACP5* alters the membrane integrity and fluidity through the alterations of FA composition (**Figure 5**).

## Conclusion

We found that *Arabidopsis* plastidial ACPs are a highly conserved family of FAS system proteins, which respond disparately to different stresses. The *atacp5* mutant plants showed increased salt sensitivity associated with a higher mortality rate, weaker growth and lower FW when compared with wild-type plants. By contrast, *AtACP5* OE lines performed better than the wild-type in each aspect. The primary difference between OE lines and wild-type plants were cytosolic Na^+^ concentration and FA composition, which indicates an involvement of *AtACP5* in the homeostasis of FAs. These results suggest that the modification of FAs contribute to the maintenance of membrane integrity, which might lead to improved salt tolerance. The findings presented here will facilitate the understanding of the relationship between cellular microenvironment and ever-changing environmental conditions.

## Author Contributions

PL designed and supervised this study; RS supervised this study; JH, CX, HW, and LW performed the experiments; WS polished the writing.

## Conflict of Interest Statement

The authors declare that the research was conducted in the absence of any commercial or financial relationships that could be construed as a potential conflict of interest.

## Footnotes

1 http://Arabidopsis.info/StockInfo?NASC_id=611501
