# Supplementary material for: Genes of ACYL CARRIER PROTEIN Family Show Different Expression Profiles and Overexpression of ACYL CARRIER PROTEIN 5 Modulates Fatty Acid Composition and Enhances Salt Stress Tolerance in Arabidopsis
Source: Front Plant Sci. 2017 Jun 8;8:987. doi: 10.3389/fpls.2017.00987 (PMC5463277; doi:10.3389/fpls.2017.00987)
Supplement: Supplementary file 1 [file Data_Sheet_1.docx]

Supplementary Material

**Genes of ACYL CARRIER PROTEIN Family Show Different Expression Profiles and Overexpression of ACYL CARRIER PROTEIN 5 Modulates Fatty Acid Composition and Enhances Salt Stress Tolerance in *Arabidopsis***

Jiexue Huang^1,2^, Caiwen Xue^1,2^ _,_ Han Wang^1^, Lisai Wang^1,2^, Wolfgang Schmidt^3^, Renfang Shen^1^, Ping Lan^1*^

*** Correspondence:** Ping Lan: [plan@issas.ac.cn](mailto:plan@issas.ac.cn)

**Supplementary Table 1 qRT-PCR Primers**

**

**

**Supple Table 2 BLAST of AtACPs**


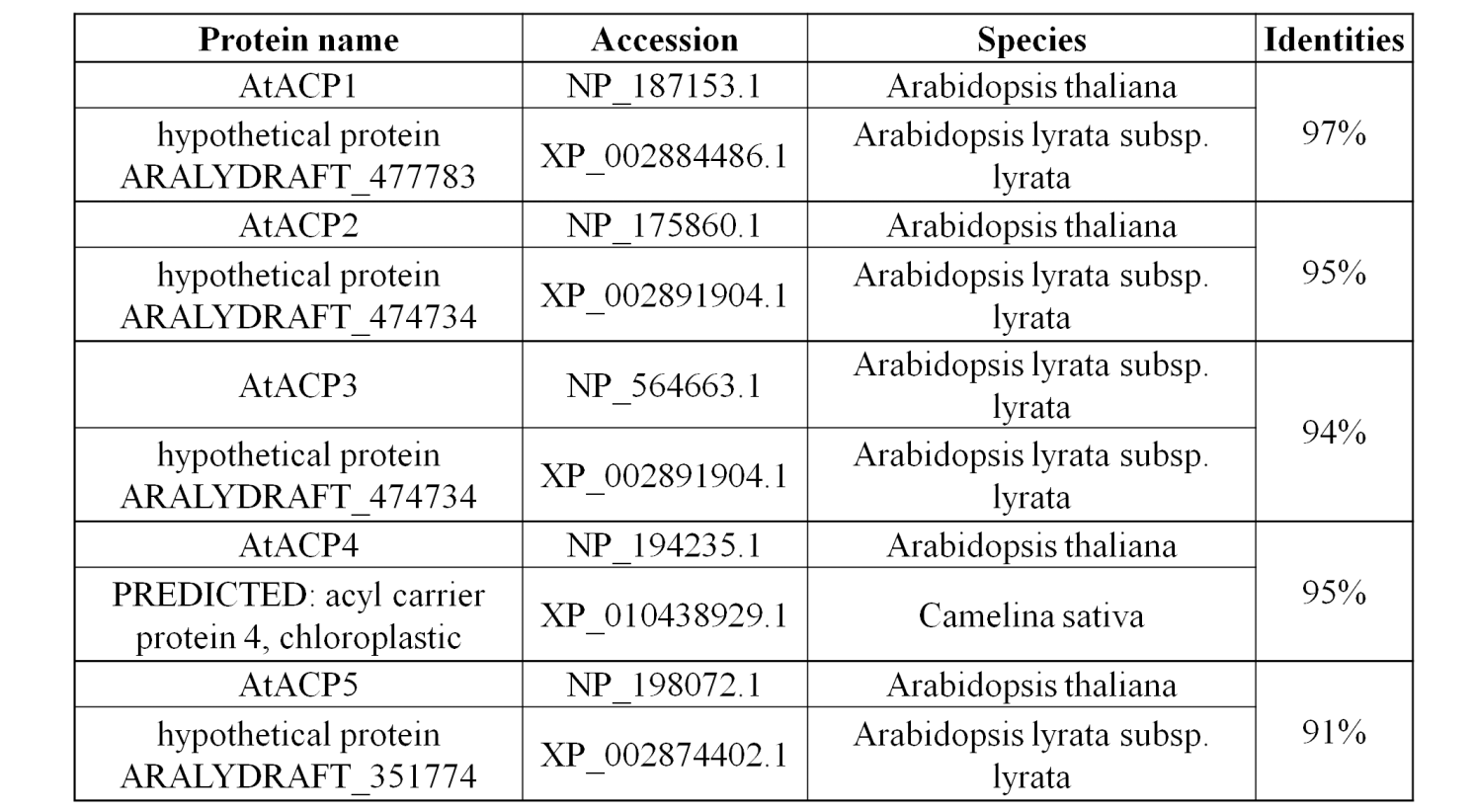


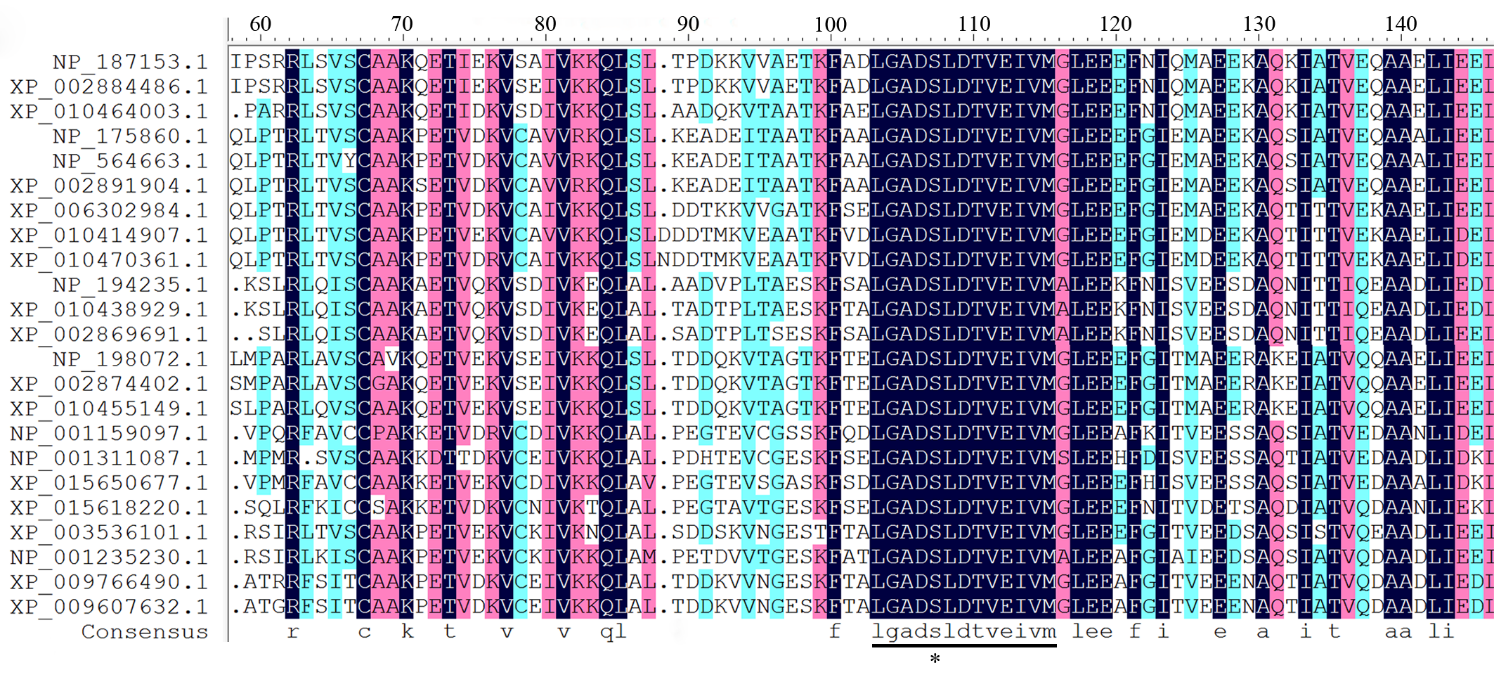
**Supplementary Figure 1: The C-terminal sequence of *Arabidopsis* plastid AtACPs is highly conserved.**

Several protein sequences with high similarity related to *Arabidopsis* plastid AtACP1-5 were retrieved using the NCBI protein BLAST (https://blast.ncbi.nlm.nih.gov/Blast.cgi). These were aligned using DNAMAN. The highly conserved region near the serine residue which the co-factor 4’-phosphopantetheine attached to was shown. The highly conserved region was indicated by underlines, and * indicated the serine residue. Accession numbers for each sequence were: NP_187153.1 AtACP1 [*Arabidopsis thaliana*]; XP_002884486.1 [*Arabidopsis lyrata subsp. lyrata*]; XP_010464003.1 [*Camelina sativa*]; NP_175860.1 AtACP2 [*Arabidopsis thaliana*]; NP_564663.1 AtACP3 [*Arabidopsis thaliana*]; XP_002891904.1 [*Arabidopsis lyrata subsp. lyrata*]; XP_006302984.1 [*Capsella rubella*]; XP_010414907.1 [*Camelina sativa*]; XP_010470361.1 [*Camelina sativa*]; NP_194235.1 AtACP4 [*Arabidopsis thaliana*]; XP_010438929.1 [*Camelina sativa*]; XP_002869691.1 [*Arabidopsis lyrata subsp. lyrata*]; NP_198072.1 AtACP5 [*Arabidopsis thaliana*]; XP_002874402.1 [*Arabidopsis lyrata subsp. lyrata*]; XP_010455149.1 [*Camelina sativa*]; NP_001159097.1 [*Zea mays*]; NP_001311087.1 [*Zea mays*]; XP_015650677.1 [*Oryza sativa Japonica Group*]; XP_015618220.1 [*Oryza sativa Japonica Group*]; P_003536101.1 [*Glycine max*]; NP_001235230.1 [*Glycine max*]; XP_009766490.1 [*Nicotiana sylvestris*]; XP_009607632.1 [*Nicotiana tomentosiformis*]


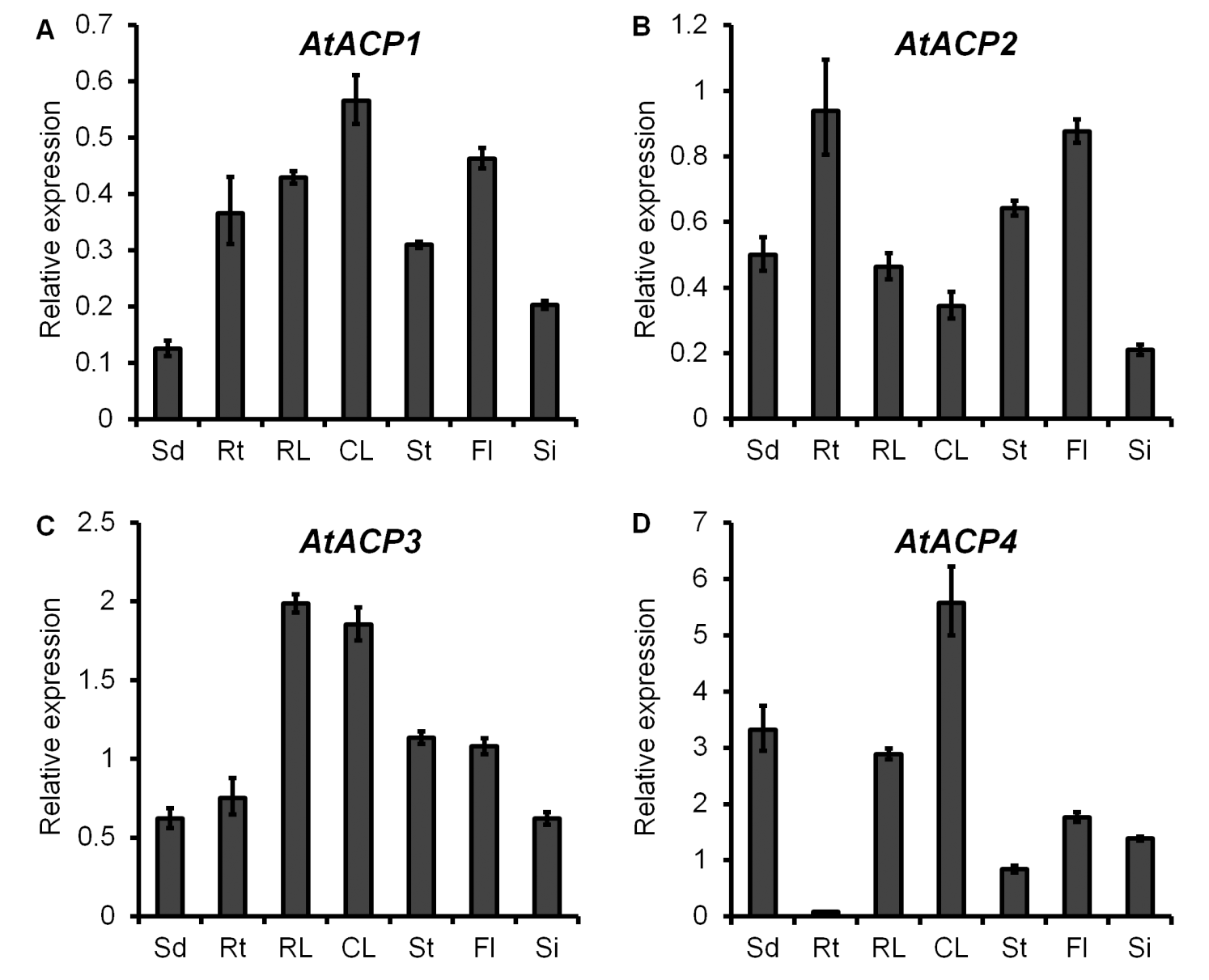


**Supplementary Figure 2. Tissues expression analyses of *AtACP1-4* in wild-type Col-0 plants.**

The qRT-PCR analyses of 4 *AtACPs* transcripts. (A) *AtACP1*, (B) *AtACP2*, (C) *AtACP3*, (D) *AtACP4*. Total RNA was extracted from 7-day-old seedlings (Sd), roots of 14-day-old seedlings grown on ES agar media (Rt), and rosette leaves (RL), cauline leaves (CL), stems (St), flowers (Fl), and young siliques (Si) of 5-week-old plants grown on nutrition soil. Expression values were calculated using 2^-ΔCT^ method with *TUBULIN ALPHA-3* (*TUA3*, At5g19770) as endogenous control. Data represents the average of three independent experiments ± SD.


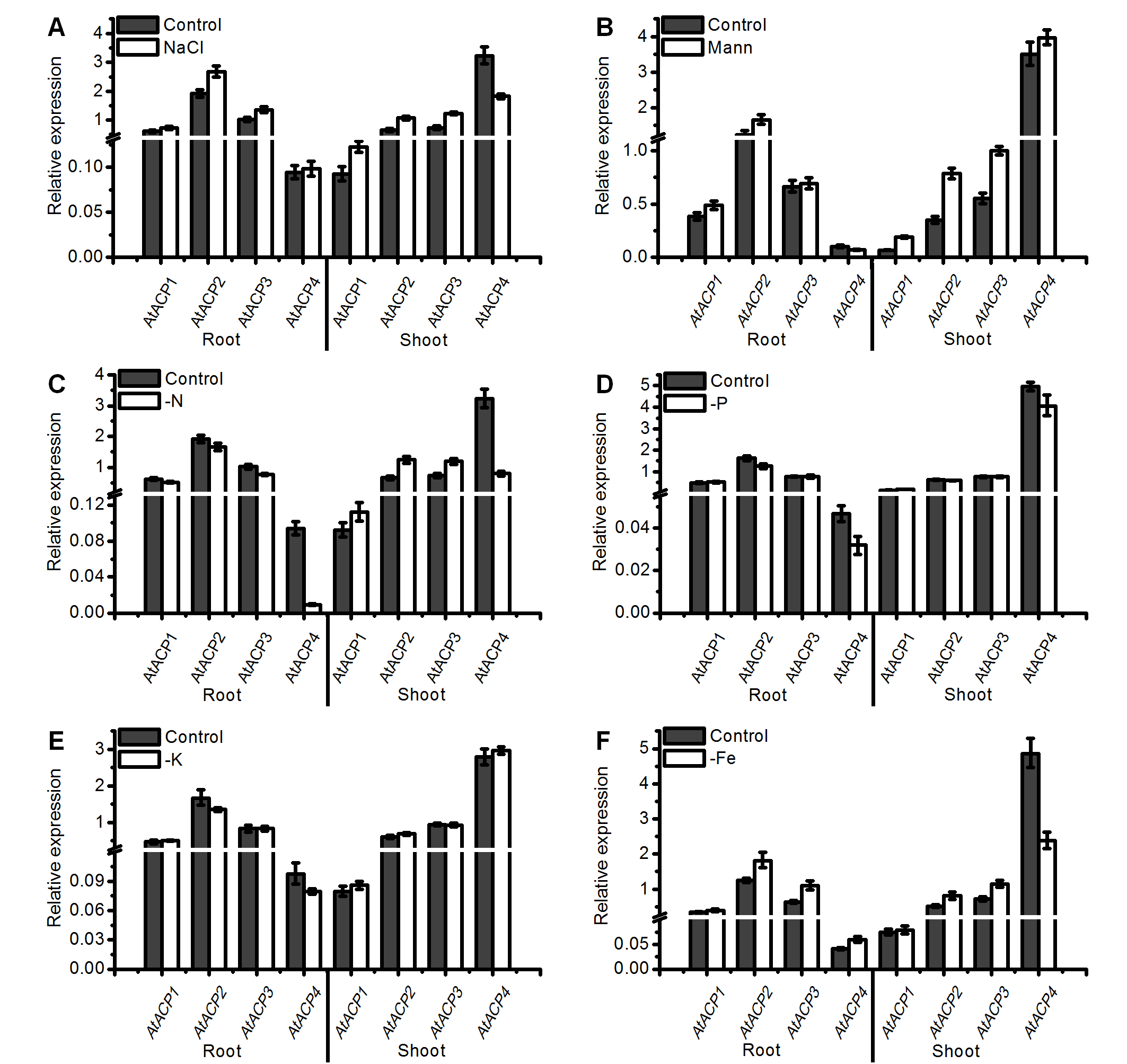


**Supplementary Figure 3. Expression analyses of *AtACP1-4* in wild-type Col-0 plants under different stresses.**

The qRT-PCR analyses of four *AtACPs* upon treatment with different stresses, including (A) 150 mM NaCl, (B) 300 mM mannitol, (C) nitrogen-, (D) phosphorus-, (E) potassium- and (F) iron-deficiency. Ten-day-old seedlings were grown on ES media and total RNA was extracted from shoots and roots after 3-day-treatment. Expression values were calculated using 2^-ΔCT^ method with *TUA3* as endogenous control. Data represents the average of three independent experiments ± SD.

**
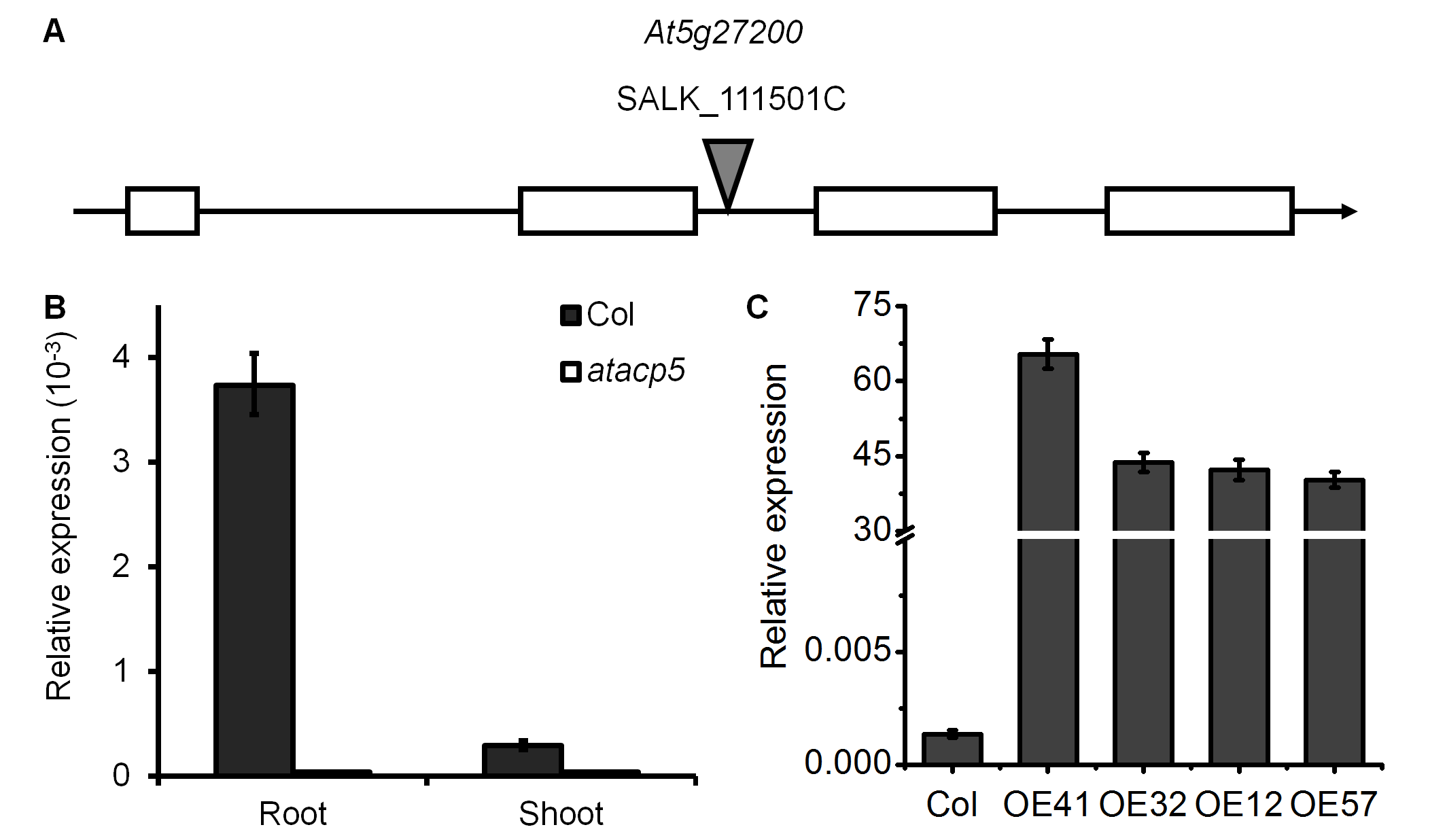
Supplementary Figure 4. Analysis of the Expression of *AtACP5* in the wild-type Col-0 plants, T-DNA insert mutant *atacp5*, and the *35S::AtACP5* overexpressing transgenic lines.**

(A). Schematic representation of the *AtACP5* (At5g27200) gene model and the mutant alleles. White rectangles represent exons, solid lines represent introns and intergenic regions, and gray triangles represent a T-DNA insertion for SALK_111501C. Expression levels of *AtACP5* in the mutant *atacp5* (B) and OE lines (C), respectively. Total RNA was extracted from 10-day-old seedlings. Expression values were calculated using 2^-ΔCT^ method and *TUA3* as endogenous control. Data represents the average of three independent experiments ± SD.


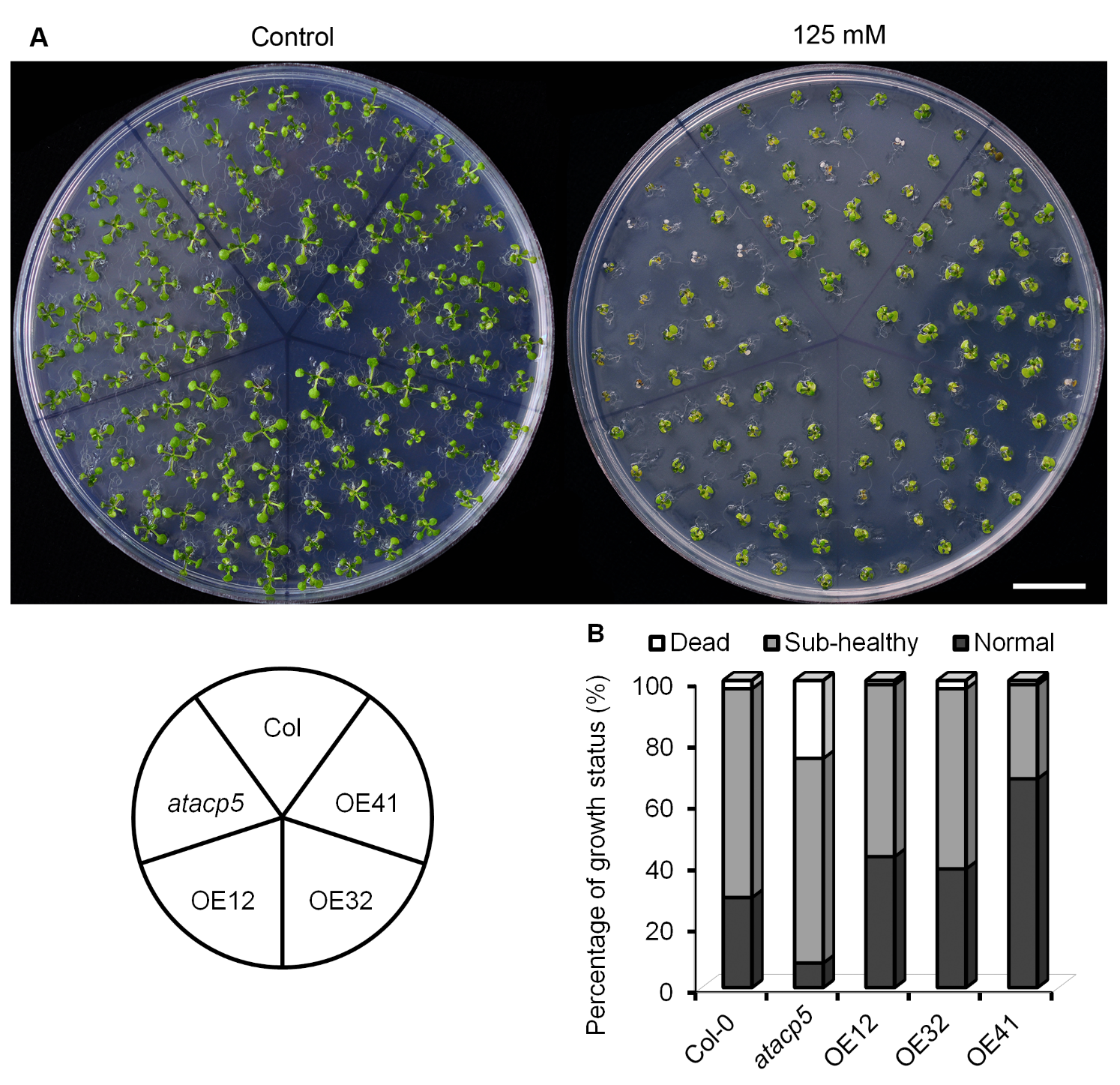


**Supplementary Figure 5. NaCl hypersensitive phenotypes of the *atacp5* mutant.**

(A). Plates with 5-day-old seedlings of Col-0, *atacp5*, OE12, OE32 and OE41 transferred to ES media supplemented with 0 mM (control), 125 mM NaCl for another 9 days. (B). Growth status and survival rate of the salt-stressed seedlings. For each treatment, three plates each containing 25 seedlings for each genotype were scored. Black column represents percentage of normal seedlings, dark grey column represents percentage of sub-healthy ones with yellowish cotyledons and white column represents percentage of white and dead seedlings. Scale bar = 2 cm.


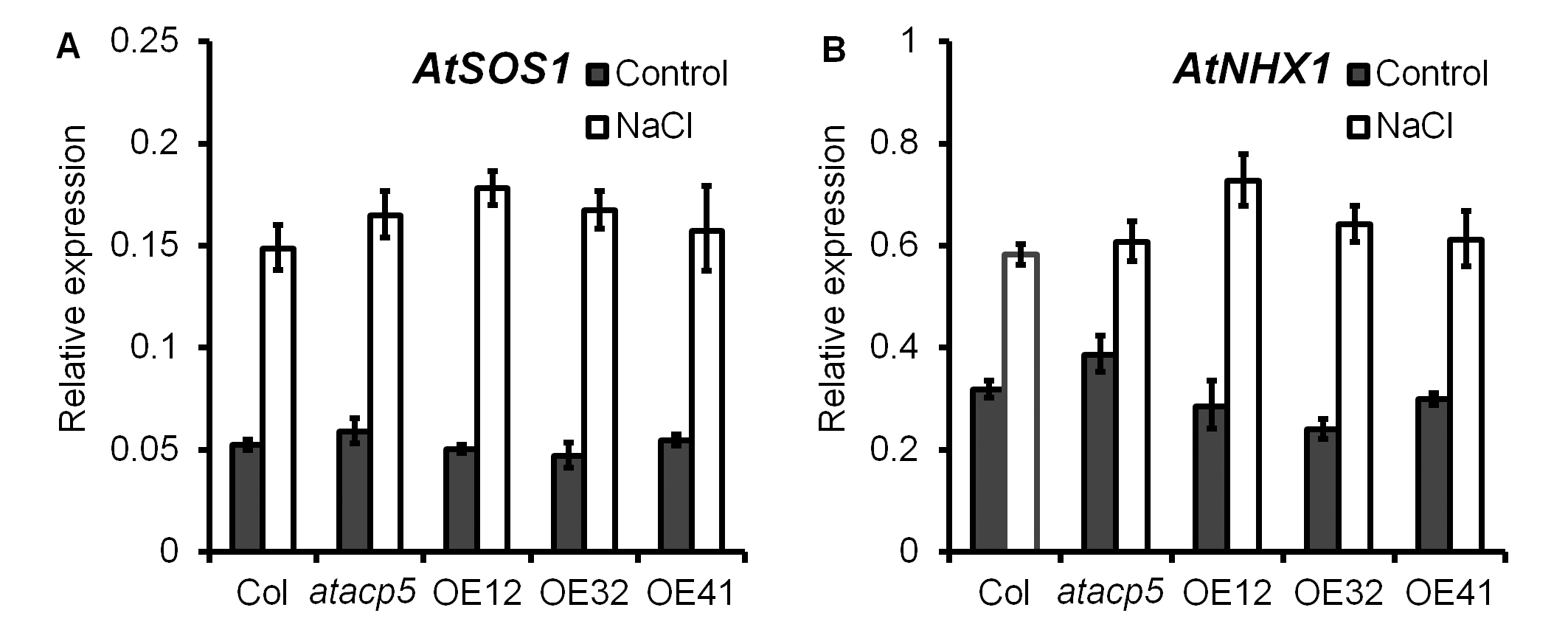


**Supplementary Figure** **6. The expression levels of genes involved in Na^+^ homeostasis.**

qRT-PCR analyses the expression of *AtSOS1* (At2g01980) (A) and *AtNHX1* (At5g27150) (B) in Col-0, *atacp5*, 3 OE lines upon NaCl (150 mM) treatment. Ten-day-old seedlings were grown on ES media and total RNA was extracted from roots after 3-day-treatment. Expression values were calculated using 2^-ΔCT^ method with *TUA3* as endogenous control. Data represents the average of three independent experiments ± SD.
